# Chemokines as key mediators in RIPI: pathophysiology and translational potential

**DOI:** 10.3389/fimmu.2025.1607447

**Published:** 2025-10-02

**Authors:** Ling Zhao, Lihua Dong, Xue Hou, Weijia Fu, Jiaying Wei, Wentong Liu, Wei Hou

**Affiliations:** ^1^ Department of Radiation Oncology & Therapy, The First Hospital of Jilin University, Changchun, Jinlin, China; ^2^ Jilin Provincial Key Laboratory of Radiation Oncology & Therapy, The First Hospital of Jilin University, Changchun, China; ^3^ NHC Key Laboratory of Radiobiology, School of Public Health, Jilin University, Changchun, China

**Keywords:** radiation-induced pulmonary injury (RIPI), chemokines, biomarker, inflammatory responses, macrophages

## Abstract

Radiation-induced pulmonary injury (RIPI) is a common adverse effect following thoracic radiotherapy (RT), and immune-related responses play a pivotal role in the pathogenesis of RIPI. Chemokines are important components of the human immune system which could modulate inflammatory responses. Their levels fluctuate following radiation. These chemokines recruit relevant immune cells, such as macrophages and lymphocytes, and induce lung inflammatory responses. In addition to early-stage inflammation, chemokines are also associated with radiation-induced pulmonary fibrosis (RIPF) at a late stage and can augment the risk of post-radiation lung metastasis. Because of the correlation between chemokines and RIPI, chemokines may be useful for RIPI diagnosis and treatment. This review aims to summarize the alterations of the levels of different chemokines after radiation, the regulatory mechanisms, and the advancements of research on the diagnosis and treatment of RIPI by chemokines, in order to provide references for the subsequent RIPI research.

## 1 Introduction

### 1.1 The pathophysiological mechanism of RIPI

Radiation-induced pulmonary injury (RIPI) is a pathophysiological process after thoracic irradiation, mainly consisting of radiation pneumonitis (RP) at the early stage and radiation-induced pulmonary fibrosis (RIPF) at the late stage (1). It could be divided into two types: non-tumor-associated RIPI and tumor-associated RIPI. The former may be caused by accidental radiation exposure or thoracic radiotherapy (RT) for non-malignant tumor-related diseases (2, 3). The latter mainly occurs after RT for malignant tumors. As one of the most important treatments for malignant tumors, RT can control tumors locally and sometimes exert an influence on distant lesions through the abscopal effect (4–6). Radiation-induced side effects in different organ systems, including RIPI, limit the use of RT in the clinic (7). Although the advancements in precision RT techniques have facilitated a certain degree of reduction in radiation-induced toxicity through the implementation of individualized treatment approaches, RIPI is still inevitable for some patients (8, 9). Moreover, its underlying mechanisms have not been fully clarified.

The complex pathophysiological process of RIPI is closely related to the damage of lung cells caused by irradiation (10). Irradiation can directly cause damage to cellular DNA, leading to double-strand breaks. It can also cause indirect damage by ionizing water molecules to generate reactive oxygen species (ROS), which further damages biological macromolecules (11–15). Damaged cells release a variety of inflammatory mediators. These mediators recruit many immune cells, such as macrophages and neutrophils, to migrate to the lungs, triggering inflammatory responses. This process is accompanied by the activation of multiple signaling pathways, which amplify the inflammatory response and exacerbate lung injury (12, 16–18). And persistent chronic inflammation can lead to the remodeling of the extracellular matrix (ECM). It can induce epithelial-mesenchymal transition (EMT) and activate fibroblasts to transform into myofibroblasts, which secrete a large amount of collagen and fibronectin. These results in the excessive deposition of the ECM, leading to the formation of pulmonary fibrosis (19, 20).

### 1.2 Chemokines participate in the development of RIPI

As important components of the immune system, chemokines are involved in various pathophysiological processes such as inflammatory responses, wound healing, and tumor progression (21–23). And as early as 1998, Johnston et al. demonstrated that the chemotaxis of inflammatory cells in response to different chemokines was involved in the pathophysiological process of RP, and some of them were also involved in advanced pulmonary fibrosis (24). These suggest that the role of chemokines in RIPI cannot be underestimated.

In recent years, there have been more studies on the relationship between chemokines and RIPI. Levels of chemokines change in the radiation-induced damage of multiple organs (25, 26). According to Zhang et al., *PTEN*, *AKT1*, *PT53*, *NOTCH1*, and *SIRT1* genes are targets of sodium butyrate (NaB) that protect against RIPI in non-small cell lung cancer (NSCLC) patients, with the former being positively correlated with chemokines and their receptors and the latter four being negatively correlated (27). And in the early stages post-irradiation, damage was dominated by parenchymal cells, with macrophages and lymphocytes being the main cell types recruited by some pro-inflammatory cytokines and chemokines (28). These illustrate the high probability that chemokines and their receptors are involved in the development of RIPI. And the role of chemokines may differ at different stages.

Most of the current studies have focused on observing changes in chemokine levels after irradiation and investigating the correlation between changes in their levels and the development of RIPI (29, 30). Researchers have observed the types of inflammatory cells recruited by chemokines after irradiation and the regulatory role of chemokines in this process (28, 31). In addition, it has also been found that chemokine levels fluctuate after RT and are associated with the pathophysiological processes of tumors. Therefore, they are sometimes used as detection indicators (32). In advanced NSCLC patients undergoing palliative thoracic RT, chemokine levels change and correlate with tumor metabolic burden (33). On this basis, some studies have also attempted to apply chemokines to the diagnosis and treatment of RIPI (34, 35).

However, some studies did not conduct dynamic observations on the levels of chemokines. Chemokines are involved in the entire process of RIPI, and the lack of continuous dynamic observations makes it impossible to clarify the characteristics of chemokines’ effects at different stages (36, 37). Meanwhile, the modeling methods for RIPI animal models, the cell lines selected in cell experiments, and the set detection time points are also significantly different. This has increased the difficulty of conducting comparative analyses of relevant studies. The limitation of clinical samples also affects the progress of research.

Currently, there is a lack of reviews that deeply explore the relationship between chemokines and RIPI and systematically elaborate on the distinct roles of chemokines in tumor-related RIPI and non-tumor-related RIPI. This review summarizes the dynamic changes of chemokines at various stages of RIPI, the relevant mechanisms, and potential future application directions, aiming to provide references for the research on chemokines in the field of RIPI.

## 2 Chemokines in RIPI: changes, characteristics, and association with RIPI

C-C motif chemokine ligand 2 (CCL2), a chemokine, primarily recruits monocytes to participate in inflammatory responses (38, 39). Owing to its functional characteristics, it was designated as monocyte chemoattractant protein 1 (MCP-1) in the early stage (40).

During the inflammatory phase of RIPI, the expression of CCL2 is rapidly upregulated within a few days after IR. It recruits monocytes/macrophages, promotes their polarization toward the M1 (pro-inflammatory) phenotype, and enhances the inflammatory response (16, 37, 41). During the fibrotic phase, CCL2 remains high expression (42). This sustained high expression drives macrophages to polarize toward the M2 (pro-fibrotic) phenotype, which then secretes factors such as TGF-β. These factors activate fibroblasts, thereby promoting collagen deposition and pulmonary fibrosis (43, 44).

In tumor-associated RIPI, during the inflammatory phase, CCL2 can upregulate inflammatory factors like IL-1β to activate tumor-associated macrophages (TAMs), forming a pro-metastatic inflammatory microenvironment (45, 46). During the fibrotic phase, the CCL2-CCR2 axis further recruits immunosuppressive cells, inhibits anti-tumor immunity, and promotes tumor progression and immune suppression (47–49).

Overall, the dynamic changes of CCL2 during the progression of tumor-associated RIPI and non-tumor-associated RIPI are similar, with both showing sustained high expression. However, in tumor-associated RIPI, CCL2 additionally exerts a role in promoting tumor progression.

The C-X-C motif chemokine ligand 12 (CXCL12)/CXCR4 axis may be involved in post-radiation damage in several organs such as radiation-induced brain necrosis. In this process, CXCL12 exerts its function as a downstream factor of HIF-1α, which can regulate not only the expression of CXCL12 but also the expression of CXCR4 (50). In RIPI, the level changes of CXCL12 exhibit a similar trend to those of CCL2, and it also plays a role in promoting RIPI.

CXCL12 exhibits a significant increase in expression during the progression of both RP and RIPF. It can initiate inflammatory responses and promote fibrosis by facilitating the migration of CXCR4^+^ bone marrow-derived mesenchymal stem cells (MSCs), fibroblasts, inflammatory cells, and other such cells to the damaged lung tissue (51, 52). In tumor-associated RIPI, the upregulation of CXCL12 can promote peritumoral tissue fibrosis, enhance the survival and immune escape of tumor cells, and reduce the infiltration of CD8^+^ T cells, ultimately leading to the impairment of antitumor immunity (53, 54).

In RIPI, the expression of CCL22 in alveolar epithelial cells is elevated. This promotes the migration of CCR4^+^ dendritic cells (DCs) to damaged tissues, induces the formation of immune-tolerant DCs, and facilitates the proliferation of Tregs, inhibiting the early inflammatory response. Currently, there is no definitive research on the level of CCL22 during the RIPF stage. However, if CCL22 continuously participates in immune regulation, maintains Tregs’ activity and immune suppression, it may potentially promote the progression of fibrosis (55). Furthermore, unlike non-tumor-associated RIPI, TAMs in TME can also secrete CCL22 to regulate immune tolerance in tumor-associated RIPI, which may potentially promote tumor escape (56).

Currently, there is limited research on the expression level and role of CCL3 in RIPI. Existing studies support that the level of CCL3 is increased in both RP and RIPF stages. Moreover, CCL3 recruits immune cells via the CCL3/CCR1 axis, enhances inflammatory responses, promotes fibroblast activation and collagen deposition, and drives the progression of pulmonary fibrosis (29, 57).

CXCL8, as a chemokine which can regulate neutrophils, has also been studied in RIPI. Its level is upregulated during the RP stage and remains highly expressed during the RIPF stage (12). CXCL8 can promote pulmonary fibrosis together with cytokines such as TGF-β, and may also be associated with processes like tumor immune escape in tumor-related RIPI (58).

The expression of CCL5 increases in the RP stage and becomes even stronger in the RIPF stage. In the RP stage, CCL5 can recruit immune cells to enhance the inflammatory response and tissue damage (59). In the RIPF stage, it can promote EMT and promote the progression of lung fibrosis (60). In tumor-related RIPI, the increased expression of CCL5 may promote tumor colonization and metastasis (61).

Chemokine KC increases rapidly in the early stage of post-radiation lung injury and then gradually decreases, and is involved in the early acute inflammatory response. It may increase again in the RIPF stage, but there is currently no sufficient evidence to indicate its relationship with RIPF. In tumor-related RIPI, its level is affected by the TME and the use of combination therapy (62–64).

CXCL10 increases significantly in the RP stage, recruits immune cells, and participates in the acute inflammatory response. In the RIPF stage, CXCL10 maintains chronic inflammation and may be associated with RIPF, but this still requires further research (10, 65). In tumor-related RIPI, while participating in the inflammatory response and causing tissue damage, CXCL10 can also recruit CD8^+^ T cells and participate in anti-tumor immunity (66).

Generally, these chemokines mentioned above are mostly upregulated in the RP stage and persistently highly expressed in the RIPF stage, with their core functions involving the recruitment of immune cells, regulation of inflammation, and progression of pulmonary fibrosis. It also points out that in tumor-associated RIPI, most chemokines additionally exert a role in promoting tumor progression, while a few can be involved in anti-tumor immunity. Besides, another important aspect is the sources of these chemokines in RIPI, and we have also made a brief summary of this part (**Table 1**).

**Table 1:** The sources and roles of chemokines in non-tumor related RIPI.

Chemokine	Cell source	Method	Sample	Role	Reference
CCL2	macrophages, lung epithelial cells, endothelial cells	10x Genomics scRNA-seq, q-PCR, ELISA, IHC	lung tissue, BALF, peripheral blood	regulate macrophage polarization, participate in the inflammatory response, and promote RIPI	(16, 18, 133)
CXCL12	fibroblasts, mesenchymal stem cells	ELISA, flow cytometry, RNA-seq, IHC	serum, lung tissue, BALF	promote the migration of inflammatory cells and mesenchymal stem cells, exacerbate lung fibrosis, and regulate the immune microenvironment	(78, 134, 135)
CXCL8	macrophages, lung epithelial cells, endothelial cells	q-PCR, ELISA, IHC	serum, lung tissue, BALF	promote neutrophil infiltration, Amplify inflammation, and Exacerbate lung injury	(2, 136, 137)
CCL22	macrophages, lung epithelial cells	q-PCR, ELISA, IHC	lung tissue, BALF	recruit immune cells, regulate immune tolerance, and promote fibrosis	(67, 138, 139)
CCL3	monocytes/macrophages	q-PCR, ELISA, IHC	serum, lung tissue, BALF	recruit inflammatory cells and promote pulmonary fibrosis	(29, 57)
CCL5	mesenchymal stem cells, lung epithelial cells, T cells, and B cells	10x Genomics scRNA-seq, q-PCR, ELISA, IHC	lung tissue, BALF	recruit immune cells, promote T cell activation, and promote pulmonary fibrosis	(45, 59–61, 140)
CXCL10	lung epithelial cells, endothelial cells, dendritic cells	q-PCR, ELISA, IHC	serum, lung tissue, BALF	promote immune cell recruitment and inflammatory response, and exacerbate lung injury	(65, 66, 141)

## 3 Regulatory mechanisms of chemokines in RIPI

Chemokines, as one of the key factors involved in RIPI, undergo regulation of multiple mechanisms. Initially, radiation induces pathological damage to tissue cells, thereby upregulating chemokine expression levels. Alveolar epithelial cells, one of the main sources of chemokines, significantly upregulate chemokine expression after sustaining radiation-induced injury. They secrete large amounts of chemokines, including CCL2 and those of the CXC family, and then recruit inflammatory cells such as dendritic cells and T cells to infiltrate (67, 68). Damaged monocytes/macrophages can also secrete chemokines such as CCL2 and CCL3. Meanwhile, they are not only chemokine-producing cells but also effector cells in the inflammatory response, and can amplify the inflammatory response by synergizing with chemokines and cytokines like IL-1β (17, 37). Even tumor cells after being irradiated can increase the release of chemokines (69). In-depth studies have revealed that this phenomenon is regulated by multiple signaling pathways.

Angiotensin-Converting Enzyme (ACE) may be involved in the pathogenesis of RIPI by regulating the ACE/type 1 angiotensin receptor (AGTR1)/NADPH oxidase 2 (NOX2) pathway and the formation of reactive oxygen species (ROS). Since with ACE inhibitor treatment, the levels of CCL2 and CCL3 in the bronchoalveolar lavage fluid(BALF) of rats with RP returned to baseline levels, and the mRNA and protein expression of CCL2 in THP-1 cells were both reduced, it is considered that ACE is highly likely an upstream regulator of CCL2 and CCL3 in RIPI (70). Moreover, the levels of Angiopoietin II (AngII) and aldosterone, downstream effector molecules of ACE, were also significantly increased in rats with RIPI and persisted high levels (71). As an upstream regulatory factor of CCL7, P21 can also regulate the expression of CCL7, but has no impact on the expression of other chemokines such as CCL2 (31).

Thrombopoietin mimetic (TPOm) exerts a protective effect by regulating pulmonary capillary endothelial cells (CapECs). It can direct CapECs to shift from radiation-induced highly activated phenotypes to homeostatic regulatory phenotypes. Meanwhile, TPOm upregulates pro-vascular homeostasis pathways such as PI and GTPase, downregulates pro-inflammatory pathways including TNF superfamily and leukocyte adhesion, and inhibits the expression of heat shock protein Hsp70, thereby alleviating endothelial cell stress injury. During this process, TPOm significantly inhibits the expression of CCL2 and KC, reducing neutrophil infiltration. This further indicates that CCL2 and KC are involved in the inflammatory phase of RIPI (15).

Although relevant research on chemokines participating in RIPI as SASP factors is few, it is known that IL-1β, which is involved in RIPI as a SASP factor, can further promote RIPF by regulating CCL2. Radiation induces bone marrow-derived immune cells to produce IL-1β, which binds to IL-1 receptors (IL-1R) on the surface of lung-resident cells. It induces the production of CCL2, recruits CCR2^+^ monocytes to migrate to lung tissue and differentiate into macrophages (36). Moreover, as the upstream factor of IL-1β, the activated NOD-like receptor pyrin domain-containing protein 3 (NLRP3) after radiation can promote the expression of IL-1β, facilitating pyroptosis. And the increased heat shock protein 27 (HSP27) after RT can act on inhibitor of nuclear factor kappa-B alpha (IκBα), activating the NF-κB pathway. This activation can promote the expression of certain cytokines, such as IL-1β, enhances EMT, and contributes to the progression of RIPF (20, 72, 73).

Additionally, the cGAS-STING signaling pathway is associated with the occurrence and development of RIPI. After radiation, the increased amount of double-stranded DNA (dsDNA) activates the cGAS-STING pathway. Once activated, STING recruits TANK-binding kinase 1 (TBK1), which promotes the activation of interferon regulatory factor 3 (IRF3) and nuclear factor kappa-light-chain-enhancer of activated B cells (NF-κB). This stimulates macrophages to secrete a variety of cytokines, including CCL2 (16, 70).

Besides these, radiation can induce changes in the expression of circular RNAs (circRNAs) and microRNAs (miRNAs). These non-coding RNAs can regulate the transcription and translation of chemokines and their receptors, affecting subsequent inflammatory responses and immune cell migration (74).

Meanwhile, research on downstream regulators in RIPI has also been reported to a certain extent at present. Chemokines primarily exert their function in RIPI by recruiting various types of immune cells. CCL2 can recruit macrophages, participate in forming the signals of the microenvironment, and regulates the phenotypes of relevant immune cells after RT. It gradually stimulates the expression of fibrosis-related proteins, leading to RIPF (16, 70). In addition to macrophages, CD45^+^ leukocytes can also be recruited by CCL2 after RT, accompanied by a significant increase in the percentage of myelomonocytes (75). In lung epithelial cells after radiation, the released chemokine CCL7 exerts a chemotactic effect on macrophages, and this effect is positively correlated with the concentration of CCL7 (31).

In addition, during this process, some other factors are also involved in the regulation. Radiation can induce fibroblasts, macrophages, and other cells to secrete osteopontin (OPN). OPN can activate the proliferation of fibroblasts, promote collagen deposition, and exacerbate fibrosis (76, 77). Silencing CXCL12 can downregulate OPN expression, thereby alleviating the degree of fibrosis, which suggests that OPN may be a downstream factor of CXCL12 in the regulation of RIPI. Meanwhile, OPN also exerts pro-tumor effects such as recruiting immunosuppressive cells, promoting angiogenesis, and facilitating tumor metastasis. This further implies that CXCL12, which is presumably upstream of OPN, may also have a pro-tumor role in tumor-associated RIPI (78).

For human fibroblasts, CXCL12 can activate the mitogen-activated protein kinase kinase kinase 1/c-Jun N-terminal kinase (MEKK1/JNK) signaling pathway to initiate the phosphorylation of Sma and MAD related protein 3 (SMAD3), promote the translocation of SMAD3 to the cell nucleus, and mediate the recruitment of SMAD3 to the connective tissue growth factor (CTGF) promoter, thereby inducing the expression of CTGF (79).

In RIPI, macrophage-related mechanisms are also one of the key focuses of attention. Most studies suggest that in the early stage of RIPI, the process is dominated by M1-type macrophages, which secrete pro-inflammatory cytokines and ROS to exacerbate inflammation and tissue damage. In the late stage, there is an increase in M2-type macrophages, which secrete pro-fibrotic factors, promoting the progression of pulmonary fibrosis (31, 80).

M1-type macrophages generated upon LPS stimulation may be more involved in RP, whereas M2-type macrophages induced by IL-4 and IL-13 stimulation may be more associated with the development of RIPF. Specifically, M1-type macrophages possess a higher capacity to produce CCL3 compared to M2-type macrophages, while CCL3 demonstrates a stronger chemotactic effect on M2-type macrophages than on M1-type macrophages. This mechanism may be implicated in the transition of RIPI from the inflammatory phase to the fibrotic phase. Nevertheless, the study did not explore whether a cascade amplification reaction of “recruitment followed by re-secretion” exists between CCL3 and macrophages (81).

In addition, lung epithelial cells exposed to ionizing radiation secrete chemokines such as CCL2 and CCL4. These chemokines can recruit macrophages to the site of lung injury, and the recruited macrophages are predominantly M2-type that express Arg-1 and CD206. M2-type macrophages may further secrete transforming growth factor-β (TGF-β), a classic cytokine that promotes EMT (44). When CCR2 expression is absent in lung tissue, the proportion of resident macrophages with an alternative activation phenotype (M2) decreases (36). And the presence of CCR2 phenotype in lung tissue contributes to the alternative activation phenotype (CD206) of macrophages, promoting the progression of pulmonary fibrosis (36).

However, the polarization of M1 and M2 macrophages is not strictly phase-separated; instead, the two subtypes can coexist dynamically. Furthermore, their polarization status is regulated by multiple signaling pathways, while the specific conversion mechanisms and timing remain not fully clarified (16, 82, 83). Therefore, the mechanism of M1/M2 macrophage polarization in RIPI cannot be fully confirmed.

Between tumor-associated and non-tumor-associated RIPI, one of the significant differences is the participation and regulation of the tumor microenvironment (TME), which are involved during the occurrence and development of tumor-associated RIPI.

Compared with non-tumor-associated RIPI, the cell types and signaling pathways in TME may exhibit differences. Tumor cells, cancer-associated fibroblasts (CAFs), and TAMs in TME may exhibit different characteristics after radiation compared with ordinary lung tissue cells. For cancer cells, increased IL-6 levels in irradiated cells upregulate the downstream protein CCL2 24 hours after RT. This upregulation encourages macrophage migration and invasion. Blocking CCL2 will decrease the number of macrophages recruited by cancer cells after RT (45). And the NLRP3 inflammasome is mainly activated in normal lung epithelial cells, which promotes the secretion of IL-1β. This in turn activates fibroblasts, leading to pulmonary fibrosis, whereas tumor cells exhibit weaker activation of this pathway (84). For CAFs, they exhibit greater radiation resistance, enter a senescent condition, persist in survival, and further affect the tumor TME after radiation. Specifically, they secrete more pro-tumor-related signaling molecules and maintain immune suppression by releasing factors such as CXCL12 (85–89).

Radiation may exert effects on the tumor genome and TME. Stereotactic Body Radiotherapy (SBRT) induces *de novo* somatic mutations in tumor cells but does not increase the tumor mutational burden (TMB), suggesting that SBRT may function by inducing “tumor-specific neoantigens” rather than increasing the total number of mutations. Regarding the tumor immune microenvironment, SBRT enhances the diversity of the T-cell receptor (TCR), expanding the range of tumor antigens recognized by T cells, and upregulates the expression of PD-L1 (90).

Using techniques such as single-cell sequencing, studies have found that CCL-related signaling may participate in the formation of an immunosuppressive TME at the early stage after radiation. In the early stage after hypo-fractionated RT, an M2-like macrophage subset (designated as Mac_Ccl8) characterized by high CCL8 expression emerges in tumors. This macrophage subset highly expresses M2 markers, immunosuppressive genes, and phagocytosis-related genes, while it lowly expresses MHC-II antigen presentation genes and pro-inflammatory genes. It can inhibit T cell activity via immune checkpoint ligands, leading to CD8^+^T cell exhaustion. Hypo-fractionated radiotherapy causes DNA damage in tumor cells, triggering tumor cells and initial myeloid cells to secrete CCL2 and CCL7. Subsequently, the recruited monocytes differentiate into Mac_Ccl8, which further secretes CCL8. CCL8 binds to CCR1/CCR5 receptors on the surface of monocytes/macrophages, promoting their migration to tumors; meanwhile, CCL2/CCL7 enhance this migratory effect via CCR2 receptors and induce the polarized differentiation of migrated cells toward the M2 phenotype. CCL8 secreted by Mac_Ccl8 further amplifies CCL signaling, recruiting more M2-like TAMs. At the same time, Mac_Ccl8 inhibits CD8^+^T cell activity via Lgals9-Havcr2 (TIM-3) signaling, forming an immunosuppressive TME (91, 92).

Under the influence of TME, TAMs exhibit a greater tendency toward the M2 phenotype, and most of them primarily function in promoting tumor progression and fibrosis (83, 93, 94). But whether macrophages in non-tumor-associated RIPI or TAMs in tumor-associated RIPI, the current research definitions and detection criteria for their phenotypes and roles in RIPI remain inconsistent. Some studies are only based on marker expression and lack functional verification. Further evidence is still needed.

Tumor-associated neutrophils (TANs) are important mediators of the antitumor effect of RT and a key component of the TME. During the early stage after RT, the levels of CXCL1, CXCL2, and CCL5 increase significantly, mediating the recruitment of TANs. On one hand, the recruited TANs can promote the infiltration of CTLs and CD8^+^ T cells into tumors, spleens, and draining lymph nodes, enhance the activation status of CTLs, and simultaneously reduce the proportion of Tregs. G-CSF can further enhance these effects of TANs. On the other hand, TANs can generate ROS to inhibit the PI3K/Akt/Snail signaling pathway, thereby blocking the EMT of tumor cells and inducing mesenchymal-epithelial transition (MET). After neutrophil depletion, the MET effect disappears, EMT is reinitiated, and the sensitivity to RT decreases (95). Moreover, the functions of TANs are diverse. Radiation-induced injury can activate neutrophils and enhance the Notch signaling pathway. Specifically, GLUT1-mediated glucose uptake in TANs enhances their pro-tumor behaviors, thereby promoting tumor growth and radiotherapy resistance. This indicates that TANs also exhibit pro-tumor effects under certain conditions (96, 97). However, there is currently no clear conclusion about the relationship between TANs and tumor-associated RIPI.

## 4 The potential of chemokines in diagnosis and treatment

### 4.1 The possibility of chemokines for predicting and diagnosing RIPI

Since chemokines play regulatory roles in RIPI, researchers have explored their application in RIPI diagnosis. Based on the relationship between CCL2 and RIPI, CCL2 has been studied in predicting RIPI. In research by Siva et al., 12 NSCLC patients receive a treatment of 60 Gy in 30 fractions over 6 weeks, with some also receiving chemotherapy. Those with lower blood CCL2 an hour after the first RT have a greater probability of severe pulmonary toxicity (according to CTCAE standard), suggesting that CCL2 could be a predictive biomarker (98). However, Yu et al. do not discover any connection between grade ≥2 radiation pneumonitis (RP2) and CCL2 levels (99). Moreover, Eleni Gkika et al. attempted to explore the relationship between CCL18 levels and early radiation lung toxicity, expecting to find the potential of CCL18 as a biomarker. However, no correlation was demonstrated between the occurrence of radiation-induced lung toxicity and the levels of CCL18 which were elevated in fibrotic diseases. But the hypothesis that CCL18 levels increase after radiotherapy is not completely disproved, as tumor regression after radiotherapy may also lead to a decrease in CCL18, allowing its levels to remain stable on the surface (100).

In addition to biomarkers, some studies have attempted to develop imaging probes targeting chemokine receptors for the visualization of RIPI lesions using PET/CT. Different from other chemokine receptors. CXCR4 has received more attention in studies for lesion imaging, especially when it serves as a tracer probe. It can be used to construct a short-wave infrared emitting nanoprobe that can targeted-detect sub-tissue microlesions in a model of lung metastasis from breast cancer up to 10.5 mm deep, with the smallest lesions up to 18.9 cubic millimeters in size (101). [^68^Ga]Ga-Pentixafor which targeting CXCR4 could be used to assess MALT lymphoma non-invasively by PET/CT (102). Vag et al. found that although the SUV_max_ of the probe targeting CXCR4 was lower than conventional [^18^F]F-FDG, it was more than tenfold in non-small cell lung cancer than other solid tumors (103). Moreover, Lau et al. designed [^68^Ga]Ga-BL01 and [^177^Lu]Lu-BL01 targeting CXCR4 for PET detection in mice with malignant tumors. The results demonstrated excellent tumor uptake and remarkable CXCR4^+^ targeting ability. This indicated the great potential of CXCR4 for application in tumor diagnosis and internal radiation therapy (104). Targeting CCR2^+^ monocytes and macrophages by ^64^Cu-DOTA-ECL1i contributed to the visualization of fibrotic lesions in IPF patients by PET and responded to treatment effects (105).

Therefore, considering the possible involvement of the CXCL12/CXCR4 axis in the development of RIPI, Pei et al. targeted CXCR4 with [^18^F] AlF-NOTA-QHY-04 and found this novel tracer detected RIPI earlier than [^18^F] FDG. Tracer uptake by irradiated mouse lung tissue increased significantly at 6 days post-RT, reached a peak at 14 days post-RT. The SUV_max_ significantly elevated in patients who developed RP in a clinical trial and may be positively correlated with the severity of RP (34). These indicates the potential of chemokines as tracer targets for early noninvasive screening of RIPI by PET/CT.

Based on the research findings above, it is obvious that there are many challenges in the application of chemokines for diagnostic purposes remain to be addressed. The accuracy of the research which using chemokines as biomarkers for predicting or diagnosing RIPI may be questionable, due to the lack of systematic detection of the dynamic changes in chemokine levels post-irradiation. Although some chemokines exhibit a general trend of change in RIPI, fluctuations in their levels still occur during the process. Both the liver and the lungs exhibit alterations in a number of chemokines, including a decrease in CCL2, CCL8, and CCL3 one to two weeks following stereotactic ablative radiation, which are different from the previous trend (106). In addition, for patients with advanced lung cancer receiving palliative RT, serum CXCL2 and CXCL6 levels also decrease during and after receiving RT (33). This indicates that in order to use chemokines as biomarkers, it is necessary to further advance the research on the mechanisms underlying their level changes, and also requires larger sample size, prospective studies to provide evidence (107).

In addition, the insufficient specificity of chemokines as biomarkers also needs to be taken into account. Since chemokines are involved in multiple immune regulation process, it is more reliable to select several relevant chemokines or other indicators with reference value to construct a model, rather than using a single chemokine. The risk of RP2 is related to baseline levels of CCL2 and IL-8 two weeks prior to RT in NSCLC patients. By integrating with mean lung dose (MLD) and hypertension, this indicator helps to produce an RP2 prediction model that had 76.5% specificity, 80% accuracy, and 100% sensitivity (99).

Meanwhile, since genetic polymorphism affects an individual’s sensitivity to inflammatory responses and disease severity. And chemokine expression is also influenced by multiple factors such as genetics, environment, and underlying diseases, there may be significant variations in expression among different individuals, making standardization difficult to achieve (108, 109). The -353A/T and +781T/C polymorphisms of the CXCL8 gene are associated with increased CXCL8 expression and elevated cancer risk (110). In addition, CCL2-2518A/G is a common single nucleotide polymorphism (SNP) in the promoter region of the CCL2 gene. It affects transcription factor binding, thereby regulating the transcriptional activity of the gene. High-expression genotypes (e.g., G/G or A/A, depending on the disease) are associated with more intense inflammatory responses, higher disease activity, and greater tissue damage. For example, in severe acute respiratory syndrome (SARS), the high-expression G/G genotype suggests a higher disease risk. In systemic lupus erythematosus (SLE), the high-expression G/G genotype is related to more severe inflammation (111, 112). This indicates that the regulatory effect of CCL2-2518A/G may vary in the context of different diseases. However, this research area remains unexplored in RIPI and requires further investigation. No matter using chemokines as biomarkers or using chemokines in combination with PET/CT for diagnostic purposes, the cost factor also needs to be taken into account.

### 4.2 Chemokines in RIPI treatment: require further exploration

Currently, as therapeutic targets, chemokines have received attention in relevant studies on various diseases. They have the potential to be therapeutic targets due to their critical roles (39, 113). For example, CXCR4 antagonists can inhibit tumor progression and metastasis, CXCL8 inhibitors can slow down tumor progression, and CCL5 inhibitors play a role in the treatment of atherosclerosis, etc. (114–116).

In RIPI treatment, adoptive transfer of MSCs during the early post-irradiation period could restore decreased superoxide dismutase 1 (SOD1) levels and reduce vascular damage and endothelial cell loss (75). In addition to the paracrine pathway (117, 118), MSCs can also play a therapeutic role through homeostatic differentiation, immunomodulation, and exosome secretion (119). For example, miR-466f-3p in exosomes derived from murine MSCs prevent radiation-induced EMT by targeting c-MET to inhibit the AKT/GSK3β pathway (120). The amount and duration of the accumulation of MSCs at the site of injury are still challenges to be solved, and further research is required to investigate the optimal time, concentration, and route of administration of the treatment (121). Meanwhile, mesenchymal stem cells (MSCs) are also closely associated with chemokines in RIPI. Therefore, whether chemokines can also be applied to RIPI treatment remains to be further studied.

CXCR4-overexpressing human umbilical cord mesenchymal stem cells (HUMSCs) showed better effects. They accumulated more in irradiated lungs and enhanced the protective effect, manifesting as attenuated interstitial hyperplasia, interstitial-patchy congestion, and interalveolar septal thickening. In addition to this, they reduced the expression levels of CXCL12, TGF-β1, and α-SMA, while enhancing the expression of E-cadherin (35). TGF-β1 is a crucial cytokine in the development of RIPF. And the production of α-SMA indicates the differentiation of myofibroblasts (122). The reduction of both helps to resist RIPF. E-cadherin is an epithelial marker that reflects the integrity of epithelial tissue and is also frequently used in EMT-related studies (123).

Additionally, when chemokine receptors are selected as targets, it is necessary to consider that some chemokines have multiple receptors. In RIPI, chemokine ligands binding to different receptors may produce different effects. For CCL3, its receptor CCR1 exhibits synchronized changes with CCL3 after irradiation. Furthermore, blocking CCR1 to inhibit inflammation in the early stage (inflammatory phase) can also exert a protective effect on long-term pulmonary fibrosis. However, CCR5, which acts as another receptor for CCL3, exerts a protective function against RIPI. In mouse experiments, following the knockout of CCR5, both the degree of pulmonary inflammation and the level of lung tissue damage in irradiated mice were significantly increased. But whether blocking the expression of one receptor for CCL3 will lead to enhanced activity of the pathway mediated by the binding of CCL3 to its other receptor remains to be further investigated (29).

The chemokine network is complex. The ligands and receptors of chemokines can exhibit cross-interaction, and additionally, atypical chemokine receptors (ACKRs) are also involved in regulation. If a single chemokine receptor is selected as a therapeutic target, other chemokine receptors will likely be upregulated to maintain the signaling pathway, leading to the occurrence of receptor compensation effect (124–126). CXCR4 and CXCR7(also known as ACKR3) can be expressed individually or collectively. When CXCR4 is inhibited, CXCR7 can substitute to mediate CXCL12 signaling, promoting tumor cell migration, angiogenesis, and metastasis (127). Therefore, selecting chemokines as therapeutic targets for RIPI requires full consideration of the complexity of the chemokine regulatory network. While the mechanism of chemokines in RIPI is being further elucidated, multi-target combination therapy can be taken into account.

Beyond these, it is also necessary to consider whether targeting chemokines will induce immunosuppression. This question is particularly critical in tumor-associated RIPI. Some studies suggest that blocking specific chemokine pathways can reduce the infiltration of immunosuppressive cells in TME, while also enhancing the function of effector immune cells like CD8^+^ T cells. For example, blocking chemokine pathways such as CXCL12/CXCR4, CCR8, and CCL2 can reduce the infiltration of cells like Tregs, MDSCs, and M2-type macrophages in TME. This effect helps reverse immunosuppression and improve the efficacy of immunotherapy (128–132). However, current research on targeting chemokine for RIPI treatment remains relatively limited, and the effects induced by this therapeutic strategy have not yet been fully clarified. Thus, there is still a long way from truly applying chemokines to clinical diagnosis and treatment practice.

## 5 Conclusion

**Figure 1:**
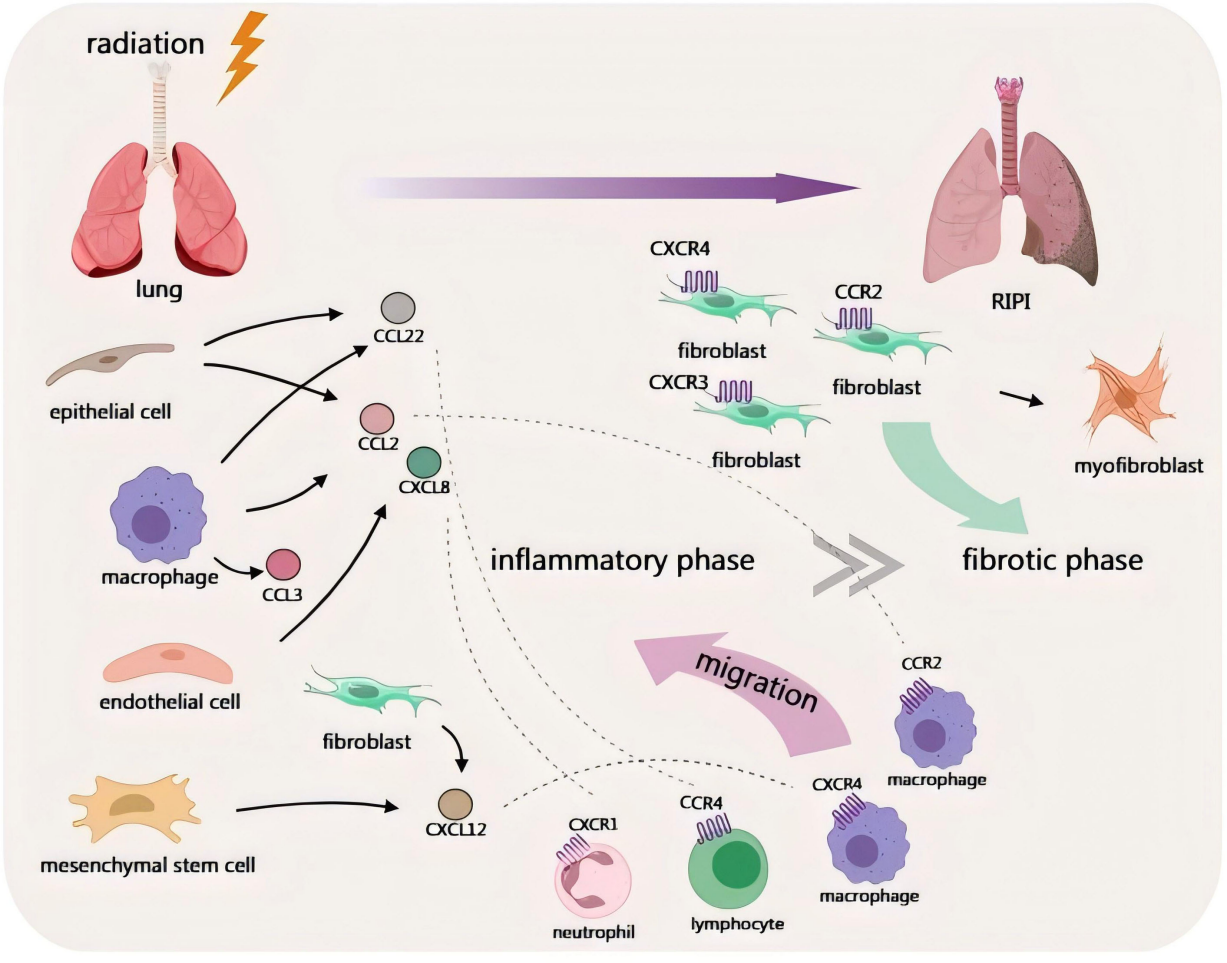
Schematic diagram of the involvement of chemokines in non-tumor-associated RIPI.

This review focuses on the role of chemokines in RIPI and systematically summarizes the regulatory role of chemokines in the pathological process of RIPI, as well as related mechanisms and explorations of translational applications. RIPI can be divided into tumor-associated and non-tumor-associated subtypes (**Figure 1**). Chemokines not only participate in lung tissue injury and repair but also are affected by TME in tumor-associated RIPI, exhibiting additional specific regulatory effects.

Mechanistically, the dynamic changes of chemokines persist throughout the entire course of RIPI, and their expression and functions are regulated by many factors. In terms of translation, chemokines show potential in the diagnosis (e.g. biomarkers, imaging targets) and treatment (e.g. targeted intervention strategies) of RIPI. However, current research still has challenges such as insufficient standardization and treatment complexity.

Future research should focus on some aspects. Firstly, promote basic research to clarify the differences in the core roles of chemokines among different RIPI subtypes. Secondly, promoting translation to optimize diagnostic tools and explore effective treatment. Finally, strengthening clinical validation to advance the practical application of chemokine-related technologies in the precision diagnosis and treatment of RIPI, in order to help to address the challenges in the clinical management of RIPI.
